# Epidemiological Context and Risk Factors Associated with the Evolution of the Coronavirus Disease (COVID-19): A Retrospective Cohort Study

**DOI:** 10.3390/healthcare10112139

**Published:** 2022-10-27

**Authors:** Leyla Hadef, Brahim Hamad, Salma Hamad, Amira Laouini, Nouri Ben Ali

**Affiliations:** 1 Department of Agronomy, Faculty of Life and Natural Sciences, University of El Oued, El Oued 39000, Algeria; 2 Laboratory of Hygiene and Animal Pathology, Institute of Veterinary Sciences, University of Tiaret, Tiaret 14000, Algeria; 3 Department of Cellular and Molecular Biology, Faculty of Life and Natural Sciences, University of El Oued, El Oued 39000, Algeria; 4 Public Institution of Neighborhood Health Saad Lamamra, El Oued 39000, Algeria

**Keywords:** COVID-19, epidemiological situation, incidence, mortality, risk factors

## Abstract

Since its initial appearance in December 2019, COVID-19 has posed a serious challenge to healthcare authorities worldwide. The purpose of the current study was to identify the epidemiological context associated with the respiratory illness propagated by the spread of COVID-19 and outline various risk factors related to its evolution in the province of Debila (Southeastern Algeria). A retrospective analysis was carried out for a cohort of 612 COVID-19 patients admitted to hospitals between March 2020 and February 2022. The results were analyzed using descriptive statistics. Further, logistic regression analysis was employed to perform the odds ratio. In gendered comparison, males were found to have a higher rate of incidence and mortality compared to females. In terms of age, individuals with advanced ages of 60 years or over were typically correlated with higher rates of incidence and mortality in comparison toindividuals below this age. Furthermore, the current research indicated that peri-urban areas were less affected that the urban regions, which had relatively significant incidence and mortality rates. The summer season was marked with the highest incidence and mortality rate in comparison with other seasons. Patients who were hospitalized, were the age of 60 or over, or characterized by comorbidity, were mainly associated with death evolution (odds ratio [OR] = 8.695; *p* = 0.000), (OR = 6.192; *p* = 0.000), and (OR = 2.538; *p* = 0.000), respectively. The study identifies an important relationship between the sanitary status of patients, hospitalization, over-age categories, and the case severity of the COVID-19 patient.

## 1. Introduction

Since having been declared a public health emergency of global concern by the World Health Organization [1], COVID-19 has undergone an explosive expansion across the globe since March 2020 [2]. As of 4 April 2022, a global case count of 491,871,560 cases with a death toll of 6,176,629 deaths has been confirmed; three countries have been marked by a significant number of cases—the United States (81,832,612 cases), India (43,029,044 cases), and Brazil (29,999,816 cases) [3]. A falling trend in terms of the effective disease regulation mechanisms may be implied by these statistics, compounded by a lack of efficient detection, a lack of awareness regarding the current epidemiological concept, and adelay in establishing policies and prevention programs during the early stages of the pandemic [4]. The virulence and infectivity of the virus, as well as population mobility, notably impact the emergence and prevalence of various viruses [5], in addition to economic activities and the social environment, which constantly interact with and respond to each other [6].

As is the case with several other regions across the globe, Algeria, Africa’s largest (2,381,740 km^2^) and tenth most populous (43,949,908 inhabitants) country, with relatively high population movement, has failed to escape the adverse effects of the pandemic [7]. Algeria is the second African country to be affected by the pandemic, with its first case having been reported on the 25 February 2020 [8]; as of April 4th this year, 265,691 cases have been recorded in Algeria, deeming it one of the most affected African countries with a death toll (6874) ranking sixth in this context, following South Africa (100,050), Tunisia (28,323), Egypt (24,417), Morocco (16,060), and Ethiopia (7504) [3]. It is rather apparent that COVID-19 has posed a major threat to sanitary authorities across the globe, highlighting the urgent need to comprehend, in a timely fashion, the epidemiological context of their corresponding populations to enact effective mechanisms that may regulate the spread of this disease [9].

Incidence, mortality, and lethality are various measures of the epidemiological context that require an assessment to efficiently manage and regulate the pandemic [10]. While intervention measures undertake a significant responsibility in this context, it is necessary for all actors to collaboratively work toward preventing the spread of the virus in a transparent and coordinated manner [2]. The increasing level of risk, and especially uncertainty caused by the COVID-19 pandemic, can contribute, on the one hand, to faster spread of the disease, and on the other hand, to great pressure for healthcare institutions. COVID-19 has rapidly become a disease associated with unbridled uncertainty with its aetiology and control, for the healthcare systems and health professionals who provide care, and among its ultimate victims, patients, and their families [11]. On the other hand, many different risk factors are associated with COVID-19 severity, such as older age, diabetes, hypertension, obesity, and male gender [10,12].

Owing to the polymorphic association between the ACE-2 receptor gene and androgen receptor genes in relation to an X chromosome-linked inheritance [13,14], the hyperandrogenic phenotype is believed to correlate with viral spread and viral load, as well as the severity of lung involvement [13]. Moreover, ACE-2 receptors, which facilitate the entry of SARS-CoV-2 within host target cells, are produced at a much lower rate in women due to their relationship with estrogen, [15] while levels of proinflammatory cytokines, such as CRP (C-reactive protein) and different interleukins, and even “cytokine storms” are increased due to the reduction intestosterone among aging men, thereby worsening the progression and severity of COVID-19 among this group [15,16].

However, there has been limited research on this particular virus in terms of the epidemiological context and clinical manifestations among patients due to its novelty and recency [17,18], specifically in terms of data on potential risk factors that contribute to Algeria, or globally. The study intends to determine the epidemiological indicators of COVID-19 with specific regard toits sociodemographic and environmental context; the research also investigates the various risk factors that may contribute to the evolution of this disease.

## 2. Materials and Methods

### 2.1. Study Area Description

Located in the southeast region of Algeria in the El Oued province, the district of Debila may be found between 33°30′23″ N and 6°56′17″ E. The area is characterized by an altitude within the range of 46–71m with an area of 2807.7 km^2^, comprising six communes with two scales of geographic occupations, four of which are considered urban areas (Hassi Khalifa, Debila, Magrane, and Hassani Abdelkrim), while Side Aoun and Trifoui represent peri-urban areas. In total, the population of this district is composed of 173,015 inhabitants, while the average population density is observed to be 61 inhabitants per km^2^. Further, the district is characterized by a significant annual growth rate. A Saharan-type climate is observed in the district, with extremely hot summers, mild winters, and sporadic rainfall during the winter months, with an annual average of 70 mm. The air temperature, wind temperature, and precipitation determine the relative humidity region, which on a monthly average range between 30% during the summers and 65% during the winters. Twenty-six degrees celcius marks the average monthly temperature of the district, represented by a large difference between both day and night, and winter and summer. Exceeding 2200 mm, the evaporation in the area is very high, with winds generally observed to be greater during fall and spring [19]. Agriculture, commerce, and animal husbandry are the primary activities that the population engages with.

### 2.2. Data Collection

The current study utilizes data on 612 patients who contracted the COVID-19 virus between March 2020 and February 2020, sourced from sanitary authorities of the Debila district. For each patient with the virus, an information sheet was filled out to collect various aspects of patient demographic information such as age, gender, address, date of consultation, disposition of the patient toward hospitalization, presence or absence of comorbidity, patient status evolution (recovery vs. death), the interval period between the consultation and confirmation of diagnosis, and the duration of stay at the hospital. Demographic information on the population per specific gender, age range, and the residential commune was sourced from the budget monitoring and programming direction of the province of El Oued. Three epidemiological indicators were explored over the course of this study, namely the incidence, mortality, and lethality rates.

The incidence rate per 100,000 habitants was calculated using the formula:

Incidence rate = [the total number of cases/the total population] × 100,000

The mortality rate per 100,000 habitants was calculated using the formula:

Mortality rate = [the total number of deaths/the total population] × 100,000

The lethalityrate was calculated using the formula:

Lethality rate = [the total number of deaths/the total number of cases] × 100

### 2.3. Statistical Analysis

Microsoft Excel^®^ 2007 and SPSS version 16.0 were used to analyze the data. A descriptive statistical analysis was performed to assess the epidemiological indicators before employing chi-squared tests to evaluate the relationship between the case evolution of COVID-19 and potential risk factors. Subsequently, the odds ratio was determined using logistic regression analysis. Case evolution (death = 0; recovery = 1) was considered the dependent variable in the context of stratified and interaction analyses, while the independent variables were represented by age range (<60 = 1; ≥60 years = 2), gender (male = 1; female = 2), patient disposition (no hospitalization = 0; hospitalization = 1), comorbidity (absence = 0; presence = 1) and the interval time (>3 days = 0; ≤3 days = 1). For all statistical analyses, the statistical significance was considered to be at the *p* level of <0.05.

## 3. Results

In the district, 612 COVID-19 cases were found with an incident and mortality rate of 353.72 per 100,000 habitants and 61.26 per 100,000 habitants (106 patients died), respectively. This may be observed in Table 1. A lethality rate of 17.32% was reached for the same district. In the study, 225 female cases (36.76%) were observed with 387 male cases (63.24%), with incident rates of 262.82 and 442.75 per 100,000 habitants, respectively, and mortality rates of 53.73 and 68.64 per 100,000 habitants, respectively. As opposed to the above statistics, the lethality rate was found to be relatively significant in females (20.44%) than in males (15.50%). Similarly, compared to younger individuals, patients aged 60 or over were characterized by a relatively high incidence rate (3128.43 vs. 199.50 per 100,000 habitants). Similarly, the mortality and lethality rates were found to be relatively high for older individuals (933.04 vs. 12.81 per 100,000 habitants) (29.82 vs. 6.42%). Relative to the peri-urban regions, urban areas were demonstrative of higher incidence and mortality rates, which were 368.13 and 64.80, as opposed to 278.96 and 42.91 per 100,000 habitants. In terms of the seasonal influences observed with regard to the spread of the disease, summers indicated a relatively high incidence (187.26 per 100,000 habitants), mortality (45.66 per 100,000 habitants), and lethality rates (24.38%). Table 1 summarizes the results regarding epidemiological indicators in the context of environmental and sociodemographic conditions.

The relationship between the disease evolution of COVID-19 and the risk factors has been depicted in Table 2. According to the findings, a significant relationship was observed between patients of ages equal or above 60 years and mortality evolution (OR = 6.192; *p* = 0.000). Further, a significant relationship was observed between a higher risk of mortality cases and COVID-19 patients with comorbidity (OR = 2.538; *p* = 0.000). Moreover, patients who stayed in hospitals for long periods were associated with a relatively higher risk of mortality compared to those who were not hospitalized at all (OR = 8.695; *p* = 0.000). Regardless, there was no relationship between interval time (OR = 1.147; *p* = 0.523) or gender (OR = 0.714; *p* = 0.119) and COVID-19 disease evolution. According to the findings, comorbidity and increased age were identified as major risk factors that may potentially affect the sanitary status of patients struggling with COVID-19 disease.

## 4. Discussion

The ongoing pandemic has garnered a significant amount of interest within academia, resulting in vast amounts of literature on the subject [20,21]. The purpose of the current study was to establish indicators associated with the ongoing pandemic within the Debila district, such as the incidence rates, mortality rates, and lethality rates. In addition, the study also evaluated various risk factors that may contribute to the critical evolution of the viral disease. Consequently, the findings from the study may prompt authorities associated with sanitation to develop efficient strategies to prevent the spread and contraction of COVID-19.

Men are considered relatively more vulnerable and increasingly exposed to the risk of mortality as a result of COVID-19, owing to the higher incidence and mortality rates found among males in our study. In line with this finding, the extant literature reports the variance in the occurrence of the virus across types of gender, generally indicating that men were relatively more affected by the disease [22,23,24]. In our study, this phenomenon may be underpinned by the relatively higher risk of exposure among men [25], as they typically work in jobs that have a higher risk of contact with other people, which facilitates the transmission of the viral disease. Furthermore, a significant relationship has been established between high-risk practices (such as smoking, disregarding rules of social distancing, and refusing to wear masks) and men, which predispose them, to a relatively great extent, to the likelihood of contracting COVID-19 disease [16,26,27]. Note that the higher mortality rate found to be among the men in this study agrees with the previous literature [10,23,24]. Drawing from a number of explanations associated with biological characteristics regarding the variances in the severity of the diseases across gender [15,16], it is important to consider the theory of relative increase vulnerability among men due to a notably significant expression of androgen receptors, which are deemed as essential in the genetic transcription of virus proteins; for example, spike protein) [28].

In contrast, adaptive and innate immune responses are encouraged by estrogens to rapidly clear pathogens, which results in relatively lower-severity symptoms and firm immune responses to vaccines among women [15,16]. In the early phase of the infection, conjoined with preferential sex-specific T cell activation, an increase in levels of proinflammatory response is observed to decline in older men, while remaining robust in aged women as well—resulting in older men being at a relatively higher risk of contracting the COVID-19 infection [16].

According to several reports, lethality rates are reported as generally high among men compared to women [10,29,30]. However, the current study revealed a higher lethality rate among females, which aligns with Joe et al. [31], who found a similar statistic (3.3% among women vs. 2.9% for men) in his India-based study. This has been chalked up to the lack of access to health services; studies have observed a difference in willingness to be admitted to the hospital across the two genders [32]. Overall, such variations in the context of COVID-19 case fatality may be attributed to the heterogeneity of data among studies, particularly in terms of sample size and demographic information such as age, health status, lifestyle, socioeconomic levels, cultural practices, effectiveness and type of sanitary services corresponding to the study country, and the individual variance across participants. For example, the difference in the average ages across the sexes (57.89 for women vs. 56.91 years for men) may have resulted in a higher lethality rate among women. Several reports have highlighted the negative engagement between the severity of COVID-19 and advanced age [22,33,34,35]. Due to a potentially weaker immune response, significantly older patients are typically more vulnerable to developing and succumbing to ARDS (acute respiratory distress syndrome) [13,33], which may be further compounded and explained by diabetes, cardiovascular conditions, hypertension, and coronary diseases, which represent comorbidities, more so among women than among men (32.4% vs. 26.9%).

Together, these factors characterize a significant inflammatory component that directly modulates the immune system of the individual, thereby increasing the these patients’ vulnerability to contracting the virus [33,35,36]. Previous studies have identified pregnancy as a significant factor that typically increases the risk of fatality due to the contraction of the COVID-19 virus [37,38]. Allotey et al. [39] conducted a meta-analysis of 77 cohort studies, which included patients who were suspected to have COVID-19 (or were confirmed to have the illness) and were pregnant; the researchers discovered that these women were less likely to show symptoms of the illness relative to women of the same age who were not pregnant, although the former characterized a higher risk for admission to intensive care units. The study also noted that these women were more likely to undergo a premature delivery, with their newborns being admitted to the neonatal intensive care unit. Overweight/obesity, increased age, and previously existing comorbidities such as diabetes and hypertension are significantly correlated witha higher risk of COVID-19 contraction with greater severity [39]. Furthermore, cardiovascular function, the respiratory system, and overall immune response may be vastly impacted by changes in physiology due to pregnancy [40].

Various works of research that are supported by global investigations of the disease generally claim the incidence rate to be relatively higher among older populations, which is in line with our study as well [41,42,43]. Such a finding may have occurred due to the weakened immune systems that older individuals generally have, which may have facilitated the transmission and severity of the COVID-19 disease [33,42]. In this context, COVID-19 patients with advanced ages were associated with a higher peak load, while vulnerability among patients infected by SARS-CoV has been linked with a high initial viral load [22,44]. In addition to a notable reduction in cell-mediated and humoral immune function, patients underwent defects in terms of the functions of their T and B lymphocytes due to their advanced ages [22]. A poor outcome would occur due to further prolonged pro-inflammatory responses and weakened control of viral replication, which takes place as a result of the overexpression of type 2 cytokines [34].

In our assessment of mortality measures and age interactions, the results obtained showed that these indicators were higher in older patients relative to younger ones, which may have taken place due to the comparatively higher percentage of comorbidities across the older age group (42.5% vs. 17.1%). This explanation is in line with previous studies, which state that comorbidities and low immune function in older patients may have been an important factor that contributes to higher mortality rates among COVID-19-infected patients [42]. Similarly, the extant literature states that mortality and lethal rates were generally found to increase with age, which aligns with the findings in this study [10,22,31,43]. Diabetes, obesity, and various cardiovascular illnesses have been linked to the variance in mortality rates [26,34]. Moreover, it must be noted that higher rates of hospitalization due to COVID-19 diseases have been notably linked to groups of older adults, and subsequently, the probability of developing severe complications among several patients who had been admitted is significant [45,46].

A significant topic that has given rise to several discussions is the impact of population density on the growth of the pandemic. Earlier scientific reports demonstrated that the coronavirus disease was primarily transmitted between people via close contact, through respiratory droplets containing the virus, which travel further when an infected individual coughs or sneezes [47,48]. Accordingly, the current study indicates that the likelihood of COVID-19 vulnerability is higher among densely populated urban areas, as the high density would increase the risk of transmission among individuals. This hypothesis is strongly supported by the significant disproportion observed across urban and peri-urban localities in terms of the population density (77.65 h/km^2^ vs. 29.75 h/km^2^, respectively). Thus, previous studies have marked population density as a potential indicator of the spread of the virus, reporting a positive relationship between the two variables [48,49].

Consequently, other studies have used this indicator as a surrogate parameter for social distancing capacity, particularly in the context of densely populated areas, implying the need for more stringent measures or implementations to ensure distancing [49,50]. Although studies across several countries have reported results regarding the interaction between the population density and incidence rate, which concurs with the findings of the current study as well [47,48,50], this association must be interpreted cautiously due to the possibility of variations being induced as a result of different factors, such as access to healthcare infrastructure, health awareness levels, connectivity among individuals, and social distancing respect policies [51]. Regardless, the notable divergence in terms of the mortality indicators observed across the regions in this study may be attributed to the higher occurrence of chronic diseases among populations within the urban areas, relative to that of peri-urban regions (83.6 vs. 16.4%, respectively). Furthermore, this supports the idea that various unhealthy lifestyle habits, such as smoking, low physical activity, and poor eating habits, may spread among areas with higher population densities, which may further cause them to be increasingly vulnerable to sanitary complications and mortality as a result of COVID-19. Further, the increase in mortality indicators among urban areas may be attributed to the disproportionate rate of patients with advanced ages therein, relative to that of lower-density regions (86.3 vs. 13.7%).

In this context, it is a well-established fact that COVID-19 severely impacts older populations that are characterized by comorbid conditions [50]. Many studies across the globe put forward a significant association between population density and mortality rates [47,48,50,52]. Likewise, the availability of health infrastructures and the household income levels in regions with high population density are primary factors that contribute to the rise in their corresponding mortality rates. Hence, it is logical to assume that individuals residing in low-density areas have poor access to healthcare and are characterized by lower household incomes, which may negatively influence the health awareness in the region; consequently, these residents may demonstrate a reluctance to seek healthcare, which may represent an underestimation of the COVID-19 mortality cases in the peri-urban regions.

In general, environmental factors can impact the transmission of COVID-19 diseases by affecting the infectivity of pathogens [53] as well as the propagation of respiratory droplets [54,55]. Similarly, our findings indicate an increased vulnerability toward and severity of the COVID-19 disease during summer, relative to other seasons. In accordance with our results, Dong et al. [56] reported that the confirmed cases of COVID-19 increased rapidly in August, potentially due to the increase of virulence during the summer season. Moreover, previous studies have confirmed this hypothesis of higher transmissibility of COVID-19 during the summer as well, noting that the number of aerosol particles during this time increase with the temperature. Since the SARS-CoV-2 virus is more persistent in low-humidity environments with high temperatures, the virus only festered and expanded further [55]. Altamimi and Ahmed [57] highlighted a significant correlation between higher levels of temperature and the incidence rate of COVID-19 cases, while other studies outlined the seasonal variations of the disease in areas characterized by larger seasonal amplitudes of environmental indicators, such as in the region selected within this study [53]. Some, however, attribute the seasonal increase in incidence rates to social habit practices unique to this season, such as social events, weddings, and circumcision parties, which are characterized by a large population density within a concentrated area, thereby increasing the risk of viral transmission. Moreover, the increased number of funeral events during this season, due to the significant number of aged patients (56.5%) and those with chronic disease complications (54.8%) in summer (relative to other seasons), results in ideal environments for the viral transmission.

Season differences in mortality and lethality rates can also be explained by the pressure in healthcare institutions, which is induced by an increase in the number of patient consultations, particularly in cases that require hospitalization and admission to the intensive care unit. In contrast with the findings of this study, other reports indicated an inverse relationship between higher average temperatures and COVID-19 mortality rates [53,58]. Such a difference in the findings may be attributed to the presence of various factors, in addition to meteorological conditions, related to geographic differences and population characteristics such as population density, age distribution, comorbidity rates, and availability of healthcare institutions, that may both individually and collectively impact the COVID-19 mortality parameters.

Our results, in terms of the risk factors related to COVID-19 case mortality, showed the absence of a significant association (*p* > 0.05) between gender and mortality evolution. While women appeared to be relatively more vulnerable to the risk of mortality, this may be attributed to the higher rate of chronic comorbidities among the females in this study. In this context, Iaccarino et al. [59] highlighted the association between higher rates of obesity and heart diseases with higher rates of admission to the intensive care unit among women infected with COVID-19. Consequently, women were more likely to undergo disease complications, resulting in higher rates of mortality, relative to males. Moreover, several other factors, such as the higher average age and pregnancy conditions, may contribute to the sex disproportion of COVID-19 mortality cases. Numerous studies have claimed that patients with advanced ages and those with immune systems that are compromised (or are suffering from chronic infections) are more likely to be susceptible to the fatality evolution of COVID-19 [22,33,34]. In addition, other reports have indicated pregnancy as a major potential risk factor in the development of COVID-19 [39,60]. In line with our findings, Zhou et al. [34] and Sousa et al. [61] found that gender was not a key factor in mortality risk among patients of various age categories. However, several studies indicate males as having had higher rates of mortality or severe infection relative to females [10,24,62,63].

Several attributed the severe clinical COVID-19 outcomes in men to the genetic fingerprint disparity, immunological mechanisms, inflammation, and sex hormone levels [16,43,64]. According to Anca et al. [64], since the X chromosome encompasses a significant number of genes that control immunity, the existence of the double (XX) chromosome in women may undertake a key role in contributing to the progression of the SARS-CoV-2 infection [64]. Further, in contrast to males, COVID-19-infected females typically produce a higher number of T cells [65]. Similarly, Zeng et al. [66] report that women are characterized by relatively higher serum concentrations of IgG antibodies during the early stages of SARS-CoV-2 infections. Moreover, as estrogen plays a key role in immune responses, research generally suggests that women have better immune responses in comparison to men [15,16,64].

In our study, advanced age was found to be a potential risk factor (*p* < 0.05) for COVID-19 mortality. Similar results were also reported by numerous authors who showed that older age is commonly associated with higher mortality risk among COVID-19 patients [10,22,31,34,43,63]. Our results indicated that among the deceased patients, 80.2% were of age 60 or above, with an odd ratio of death risk that was sixtimes the ratio for patients below this age range. This phenomenon has been associated with the increased rate of comorbid conditions among elderly patients, such as diabetes, obesity, or various cardiovascular diseases [67,68]. Moreover, the potential for ARDS (acute respiratory distress syndrome) wasfound to be high among elderly patients due to a weakened immune system and complications owing to COVID-19 infections [33]. The unsatisfactory control of viral replication and relatively more prolonged pro-inflammatory responses among older individuals as a result of age-dependent defects were found to cause a notable reduction in cell-mediated and humoral immune functions [34]. Moreover, in the context of COVID-19 in China, higher sequential organ failure, relatively higher D-dimer, and advanced age have been identified as risk factors for COVID-19-related mortality [34].

In this study, hospitalization is a factor that presents the highest risk of COVID-19 mortality and was significantly associated with an increase in the odds ratio for COVID-19-related death. In total, 91.5% (97/106 cases) of the total death cases were hospitalized for COVID-19-related reasons, which is an alarming statistic. In line with similar results across various studies, hospitalization has been significantly associated with the number of COVID-19 death cases [61,62], which may be explained by the variation in the proportion of patients with advanced age and chronic diseases, in comparison with patients who were not hospitalized. Upon closer examination, it is clear that the proportion of elderly patients within the hospitalized group was significantly higher, relative to younger patients (58.6vs. 27.2%). Similarly, the proportion of patients with comorbidities was significantly larger among the hospitalized patients, relative to non-hospitalized patients (35.0 vs. 19.1%). Likewise, numerous reports highlighted that the rate and risk of COVID-19 hospitalization were significantly higher for older individuals and those who suffer from chronic diseases [10,34,46,69]. Elderly people and patients with comorbidities represent a vulnerable population with a greater risk of mortality due to COVID-19 infections, particularly due to poor nutrition, treatment-related side effects, and their immunosuppressed status [61,62]. Consequently, these groups are highly vulnerable to potential infections and the development of severe complications, warranting admission to intensive care units and/or resulting in death [34,63,69]. Adding to this context, Li et al. [69] stated patients with the most severe COVID-19 infections demonstrated rapid progression and multiple organ dysfunctions, leading to a fatal evolution of these cases.

The current study demonstrated a significant link between patients suffering from chronic comorbid conditions and the higher risk of COVID-20 mortality, which may be attributed to the combined effect of comorbidities and age-related defects among older patients. In line with this hypothesis, it was found that of the 177 patients who have been linked to a chronic disease, 121 were of ages 60 or over (68.4%). Similarly, extant literature has identified a significant relationship between the high risk of adverse outcomes and COVID-related mortality with regularly observed comorbid conditions such as kidney disease, hypertension, cardiovascular diseases, diabetes, or respiratory conditions [22,34,36,62,63]. This observation may suggest a link between the severity of developed atypical pneumonia in COVID-19 patients and the risk of death, in addition to their interactive comorbidity traits. Earlier researchers claimed that the increased mortality risk among older COVID-19 patients with comorbidities may be attributed to their low immune function, which makes them more vulnerable to the contraction of infectious diseases [35,42].

The present study revealed that COVID-19 death cases were not significantly (*p* > 0.05) influenced by the diagnostic confirmation period. It must be noted that the confirmation of COVID-19 infection by the healthcare authorities, particularly at the beginning of the pandemic, was carried out exclusively based on the nucleic acid test. The diagnosis processes have thus been delayed frequently, owing to the pressure of the samples and the long waiting time, as itcould take several days (up to 10 days) to access laboratories that would carry out a medical analysis. In the meantime, patients with severe COVID-19 are likely to have developed immense complications oreven died of respiratory failure. This context highlights the need for urgent and timely diagnosis, suggesting that early intervention must not be delayed due to the nucleic acid test. The initiation time and the therapeutic protocol based on antibiotic drugs and systematic corticosteroid treatments have been used similarly across the two groups (survivors and non-survivors), which may explain the insignificant impact of the diagnostic confirmation on COVID-19 case evolution demonstrated within this study. Li et al. [69], in this context, found that there was no significant association (*p* > 0.05) between the onset time and the computed tomography diagnostic confirmation in the COVID-19 patients with severe and non-severe cases, a discovery that was attributed to the relatively long duration required for such a diagnostic method (a median of 4 days). Similarly, Zhou et al. [34] outlined the absence of a significant relationship between the disease onset time and the point at which several clinical outcomes are presented, which requires one to account for the delayed time of COVID-19 disease in the function of immune status among the recovering and deceased hospital patients. Subsequently, the previous authors failed to assess a significant variance both in the intervention time and in the duration of drugs utilized within the treatment of both groups.

The study had some limitations: First, the small sample sizeprobably influenced the statistical result interpretation. Moreover, the study was undertaken in a small geographic area. Further studies are needed to investigate potential risk factors of severity in patients with COVID-19 with a large sample size and in a large geographic space. Second, the information was collected as a self-report in some cases, leading to recall bias.

## 5. Conclusions

The findings in this study demonstrated higher incidence and mortality rates among men, relative to those among women, while a lower lethality rate was observed for males. Furthermore, all three indicator rates were found to increase with advancements in age, while they were found to vary across study regions and seasons, with a noted increase in urban areas (relative to peri-urban areas) and during summers (relative to other seasons). The severity of cases and COVID-19-related death were impacted by major risk factors, which, in this study, were found to be advanced age, comorbidities, and importantly, hospitalization. Hence, it is essential to develop mechanisms and strategies in order to improve upon the regulation of the disease in various populations, which shall remain particularly crucial in countries where capacities for hospital responses and intensive care remain in their developing stages.

## Figures and Tables

**Table 1:** Epidemiological indictors, sociodemographic and environmental conditions interactions (N = 612).

Variables	Variable Categories	Population Numbers	Case Numbers	Case Percentages	Incidence Rate per 100,000 Habitants	Mortality Numbers	Mortality Percentages	Mortality Rate per 100,000 Habitants	Lethality Rate (%)
Gender	Male	87,407	387	63.24	442.75	60	56.60	68.64	15.50
	Female	85,608	225	36.76	262.82	46	43.40	53.73	20.44
	Total	173,015	612	100	353.72	106	100	61.26	17.32
Age	<60 years	163,905	327	53.43	199.50	21	19.81	12.81	6.42
	≥60 years	9110	285	46.57	3128.43	85	80.19	933.04	29.82
	Total	173,015	612	100	353.72	106	100	61.26	17.32
Region type	Urban	145,055	534	87.25	368.13	94	88.68	64.80	17.60
	Periurban	27,960	78	12.75	278.96	12	11.32	42.91	15.38
	Total	173,015	612	100	353.72	106	100	61.26	17.32
Season	Winter	173,015	110	17.97	63.57	12	11.32	6.93	10.90
	Spring	173,015	150	24.51	86.69	12	11.32	6.93	8.00
	Summer	173,015	324	52.94	187.26	79	74.53	45.66	24.38
	Autumn	173,015	28	4.58	16.18	3	2.83	1.73	10.71
	Total	173,015	612	100	353.72	106	100	61.26	17.32

**Table 2:** Relationship between the COVID-19 disease evolution and the risk factors (N = 612).

Risk Factors	Death (Case Numbers/%)	Recovery (Case Numbers/%)	Odds Ratio (OR)	*p*-Value	χ^2^
Gender (malevs. female)	Male (60/15.5)	Male (327/84.5)	0.714	0.119	2.425
	Female (46/20.4)	Female (179/79.6)	1		
Age category (≥60 vs. <60 years)	≥60 years (85/29.8)	≥60 years (200/70.2)	6.192	0.000	58.239
	<60 years (21/6.4)	<60 years (306/93.6)	1		
Hospitalization (hospitalization vs. no hospitalization)	Hospitalization (97/25.7)	Hospitalization (280/74.3)	8.695	0.000	48.482
	No hospitalization (9/3.8)	No hospitalization (226/96.2)	1		
Comorbidity (with vs. without)	With comorbidity (49/27.7)	With comorbidity (128/72.3)	2.538	0.000	18.676
	Without comorbidity (57/13.1)	Without comorbidity (378/86.9)	1		
Interval time between consultation and diagnostic confirmation (>3 vs. ≤3 days)	>3 days (61/18.2)	>3 days (274/81.8)	1.147	0.523	0.408
	≤3 days (45/16.2)	≤3 days (232/83.8)	1		

## Data Availability

Not applicable.

## References

[1] World Health Organization WHO Director-General’s Statement on IHR Emergency Committee on Novel Coronavirus (2019-nCoV). https://www.who.int/dg/.

[2] Zhao Z., Li X., Liu F., Zhu G., Ma C., Wang L. (2020). Prediction of the COVID-19 spread in African countries and implications for prevention and control: A case study in South Africa, Egypt, Algeria, Nigeria, Senegal and Kenya. Sci. Total Environ..

[3] Worldometer COVID-19 Coronavirus Pandemic Update. https://www.worldometers.info/coronavirus/.

[4] Lazzerini M., Barbi E., Apicella A., Marchetti F., Cardinale F., Trobia G. (2020). Delayed access or provision of care in Italy resulting from fear of COVID-19. Lancet Child Adolesc. Health.

[5] Redding D.W., Atkinson P.M., Cunningham A.A., Iacono G.L., Moses L.M., Wood J.L.N., Jones K.E. (2019). Impacts of environmental and socio-economic factors on emergence and epidemic potential of Ebola in Africa. Nat. Commun..

[6] Zhao W., Zhang J., Meadows M.E., Liu Y., Hua T., Fu B. (2020). A systematic approach is needed to contain COVID-19 globally. Sci. Bull..

[7] Lounis M. (2021). Epdemiology of coronavirus disease 2020 (COVID-19) in Algeria. New Microbes New Infect..

[8] Algerian Health Ministry Situation Report of 25/02/2020. http://covid19.sante.gov.dz/fr/2020/02/25/25-fevrier-2020/.

[9] Xiang Y.T., Yang Y., Li W., Zhang L., Zhang Q., Cheung T., Ng C.H. (2020). Timely mental health care for the 2019 novel coronavirus outbreak is urgently needed. Lancet Psychiatry.

[10] Esmaeili E.D., Fakhari A., Naghili B., Khodamoradi F., Aziz H. (2022). Case fatality and mortality rates, socio-demographic profile, and clinical features of COVID-19 in the elderly population: A population-based registry study in Iran. J. Med. Virol..

[11] Koffman J., Gross J., Etkind S.N., Selman L. (2020). Uncertainty and COVID-19: How are we to respond?. J. R. Soc. Med..

[12] Alberca R.W., Oliveira L.M., Branco A.C.C.C., Pereira N.Z., Sato M.N. (2021). Obesity as a risk factor for COVID-19: An overview. Crit. Rev. Food Sci. Nutr..

[13] De Sousa Lima M.E., Barros L.C.M., Aragão G.F. (2020). Could autism spectrum disorders be a risk factor for COVID-19?. Med. Hypotheses.

[14] Zheng Q.L., Duan T., Jin L.P. (2020). Single-cell RNA expression profiling of ACE2 and AXL in the human maternal–Fetal interface. Reprod. Dev. Med..

[15] Haitao T., Vermunt J.V., Abeykoon J., Ghamrawi R., Gunaratne M., Jayachandran M., Narang K., Parashuram S., Suvakov S., Garovic V.D. (2020). COVID-19 and sex differences: Mechanisms and biomarkers. Mayo Clin. Proc..

[16] Takahashi T., Iwasaki A. (2021). Sex differences in immune responses. Science.

[17] Chan J.F., Yuan S., Kok K.H., To K.K., Chu H., Yang J., Xing F., Liu J., Yip C.C., Poon R.W. (2020). A familial cluster of pneumonia associated with the 2019 novel coronavirus indicating person-to-person transmission: A study of a family cluster. Lancet.

[18] Yang X., Yu Y., Xu J., Shu H., Xia J., Liu H., Wu Y., Zhang L., Yu Z., Fang M. (2020). Clinical course and outcomes of critically ill patients with SARS-CoV-2 pneumonia in Wuhan, China: A single-centered, retrospective, observational study. Lancet Respir. Med..

[19] National Office of Meteorology (2020). Monthly Reports.

[20] Buscema P.M., Torre F.D., Breda M., Massini G., Grossi E. (2020). COVID-19 in Italy and extreme data mining. Phys. A.

[21] Scabini L.F.S., Ribas L.C., Neiva M.B., Junior A.G.B., Farfan A.J.F., Bruno O.M. (2021). Social interaction layers in complex networks for the dynamical epidemic modeling of COVID-19 in Brazil. Phys. A.

[22] Albitar O., Ballouze R., Ooi J.P., Ghadzi S.M.S. (2020). Risk factors for mortality among COVID-19 patients. Diabetes Res. Clin. Pract..

[23] Jin J.M., Bai P., He W., Wu F., Liu X.F., Han D.M., Liu S., Yang J.K. (2020). Gender differences in patients with COVID-19: Focus on severity and mortality. Front. Public Health.

[24] Vahidy F.S., Pan A.P., Ahnstedt H., Munshi Y., Choi H.A., Tiruneh Y., Nasir K., Kash B.A., Andrieni J.D., McCullough L.D. (2021). Sex differences in susceptibility, severity, and outcomes of coronavirus disease 2019: Cross-sectional analysis from a diverse US metropolitan area. PLoS ONE.

[25] Su W., Qiu Z., Zhou L., Hou J., Wang Y., Huang F., Zhang Y., Jia Y., Zhou J., Liu D. (2020). Sex differences in clinical characteristics and risk factors for mortality among severe patients with COVID-19: A retrospective study. Aging.

[26] Abate B.B., Kassie A.M., Kassaw M.W., Aragie T.G., Masresha S.A. (2020). Sex difference in coronavirus disease (COVID-19): A systematic review and meta-analysis. BMJ Open.

[27] Tadiri C.P., Gisinger T., Kautzy-Willer A., Kublickiene K., Herrero M.T., Raparelli V., Pilote L., Norris C.M. (2020). GOING-FWD Consortium, The influence of sex and gender domains on COVID-19 cases and mortality. Can. Med. Assoc. J..

[28] Wambier C.G., Goren A. (2020). Severe acute respiratory syndrome coronavirus 2 SARS-COV-2 infection is likely to be androgen mediated. J. Am. Acad. Dermatol..

[29] Qian J., Zhao L., Ye R.Z., Li X.J., Liu Y.L. (2020). Age-dependent gender differences in COVID-19 in Mainland China: Comparative study. Clin. Infect. Dis..

[30] Dehingia N., Raj A. (2021). Sex differences in COVID-19 case fatality: Do we know enough?. Lancet Glob. Health.

[31] Joe W., Kumar A., Rajpal S., Mishra U.S., Subramanian S.V. (2020). Equal risk, unequal burden? Gender differentials in COVID-19 mortality in India. J. Glob. Health Sci..

[32] Kumar K., Singh A., James K.S., McDougal L., Raj A. (2020). Gender bias in hospitalization financing from borrowings, selling of assets, contribution from relatives or friends in India. Soc. Sci. Med..

[33] Wu C., Chen X., Cai Y., Xia J., Zhou X., Xu S., Huang H., Zhang L., Zhou X., Du C. (2020). Risk factors associated with acute respiratory distress syndrome and death in patients with coronavirus disease 2019 pneumonia in Wuhan, China. JAMA Intern. Med..

[34] Zhou F., Yu T., Du R., Fan G., Liu Y., Liu Z., Xiang J., Wang Y., Song B., Gu X. (2020). Clinical course and risk factors for mortality of adult in patients with COVID-19 in Wuhan, China: A retrospective cohort study. Lancet.

[35] Ya’qoub L., Elgendy I.Y., Pepine C.J. (2021). Sex and gender differences in COVID-19: More to be learned!. Am. Heart J. Plus Cardiol. Res. Pract..

[36] Li B., Yang J., Zhao F., Zhi L., Wang X., Liu L., Bi Z., Zhao Y. (2020). Prevalence and impact of cardiovascular metabolic diseases on COVID-19 in China. Clin. Res. Cardiol..

[37] Martinez-Portilla R.J., Sotiriadis A., Chatzakis C., Torres-Torres J., Espino Y., Sosa S., Sandoval-Mandujano K., Castro-Bernabe D.A., Medina-Jimenez V., Monarrez-Martin J.C. (2021). Pregnant women with SARSCoV-2 infection are at higher risk of death and pneumonia: Propensity score matched analysis of a nationwide prospective cohort (COV19Mx). Ultrasound Obstet. Gynecol..

[38] Oakes M.C., Kernberg A.S., Carter E.B., Foeller M.E., Palanisamy A., Raghuraman N., Kelly J.C. (2021). Pregnancy as a risk factor for severe coronavirus disease 2019 using standardized clinical criteria. Am. J. Obstet. Gynecol. MFM.

[39] Allotey J., Stallings E., Bonet M., Yap M., Chatterjee S., Kew T., Debenham L., Llavall A.C., Dixit A., Zhou D. (2020). Clinical manifestations, risk factors, and maternal and perinatal outcomes of coronavirus disease 2019 in pregnancy: Living systematic review and meta-analysis. BMJ.

[40] Wastnedge E.A.N., Reynolds R.M., van Boeckel S.R., Stock S.J., Denison F.C., Maybin J.A., Critchley H.O.D. (2021). Pregnancy and COVID-19. Physiol. Rev..

[41] Chen N., Zhou M., Dong X., Qu J., Gong F., Han Y., Qiu Y.M., Wang J., Liu Y., Wei Y. (2020). Epidemiological and clinical characteristics of 99 cases of 2019 novel coronavirus pneumonia in Wuhan, China: A descriptive study. Lancet.

[42] Liu K., Chen Y., Lin R., Han K. (2020). Clinical features of COVID-19 in elderly patients: A comparison with young and middle-aged patients. J. Infect..

[43] Ramírez-Soto M.C., Arroyo- Hernández H., Ortega-Cáceres G. (2021). Sex differences in the incidence, mortality, and fatality of COVID-19 in Peru. PLoS ONE.

[44] Chu C.M., Poon L.L.M., Cheng V.C.C., Chan K.S., Hung I.F.N., Wong M.M.L., Chan K.H., Leung W.S., Tang B.S.F., Chan V.L. (2004). Initial viral load and the outcomes of SARS. Can. Med. Assoc. J..

[45] Niu S., Tian S., Lou J., Kang X., Zhang L., Lian H., Zhang J. (2020). Clinical characteristics of older patients infected with COVID-19: A descriptive study. Arch. Gerontol. Geriat..

[46] Zhu X., Yuan W., Shao J., Huang K., Wang Q., Yao S., Lu W., Liu L., Fu T. (2021). Risk factors for mortality in patients over 70 years old with COVID-19 in Wuhan at the early break: Retrospective case series. BMC Infect. Dis..

[47] Zhang C.H., Schwartz G.G. (2020). Spatial disparities in coronavirus incidence and mortality in the United States: An ecological analysis as of May 2020. J. Rural Health.

[48] Martins-Filho P.R. (2021). Relationship between population density and COVID-19 incidence and mortality estimates: A county-level analysis. J. Infect. Public Health.

[49] Wong D.W.S., Li Y. (2020). Spreading of COVID-19: Density matters. PLoS ONE.

[50] Bhadra A., Mukherjee A., Sarkar K. (2021). Impact of population density on Covid-19 infected and mortality rate in India. Model. Earth Syst. Environ..

[51] Hamidi S., Sabouri S., Ewing R. (2020). Does density aggravate the COVID-19 pandemic?. J. Am. Plan. Assoc..

[52] Pascoal R., Rocha H. (2022). Population density impact on COVID-19 mortality rate: A multifractal analysis using French data. Phys. A.

[53] Liu X., Huang J., Li C., Zhao Y., Wang D., Huang Z., Yang K. (2021). The role of seasonality in the spread of COVID-19 pandemic. Environ. Res..

[54] Mittal R., Ni R., Seo J.H. (2020). The flow physics of COVID-19. J. Fluid Mech..

[55] Zhao L., Qi Y., Luzzatto-Fegiz P., Cui Y., Zhu Y. (2020). COVID-19: Effects of environmental conditions on the propagation of respiratory droplets. Nano Lett..

[56] Dong E., Du H., Gardner L. (2020). An interactive web-based dashboard to track COVID-19 in real time. Lancet Infect. Dis..

[57] Altamimi A., Ahmed A.E. (2020). Climate factors and incidence of Middle East respiratory syndrome coronavirus. J. Infect. Public Health.

[58] Christophi C.A., Sotos-Prieto M., Lan F.Y., Delgado-Velandia M., Efthymiou V., Gaviola G.C., Hadjivasilis A., Hsu Y.T., Kyprianou A., Lidoriki I. (2021). Ambient temperature and subsequent COVID-19 mortality in the OECD countries and individual United States. Sci. Rep..

[59] Iaccarino G., Grassi G., Borghi C., Carugo S., Fallo F., Ferri C., Giannattasio C., Grassi D., Letizia C., Mancusi C. (2020). Gender differences in predictors of intensive care units admission among COVID-19 patients: The results of the SARS-RAS study of the Italian society of hypertension. PLoS ONE.

[60] Phoswa W.N., Khaliq O.P. (2020). Is pregnancy a risk factor of COVID-19?. Eur. J. Obstet. Gynecol. Reprod. Biol..

[61] Sousa G.J.B., Garces T.S., Cestari V.R.F., Florêncio R.S., Moreira T.M.M., Pereira M.L.D. (2020). Mortality and survival of COVID-19. Epidemiol. Infect..

[62] Parra-Bracamonte G.M., Lopez-Villalobos N., Parra-Bracamonte F.E. (2020). Clinical characteristics and risk factors for mortality of patients with COVID-19 in a large data set from Mexico. Ann. Epidemiol..

[63] Zhang J., Wang X., Jia X., Li J., Hu K., Chen G., Wei J., Gong Z., Zhou C., Yu H. (2020). Risk factors for disease severity, unimprovement, and mortality in COVID-19 patients in Wuhan, China. Clin. Microbiol. Infect..

[64] Anca P.S., Toth P.P., Kempler P., Rizzo M. (2021). Gender differences in the battle against COVID-19: Impact of genetics, comorbidities, inflammation and lifestyle on differences in outcomes. Int. J. Clin. Pract..

[65] Takahashi T., Ellingson M.K., Wong P., Israelow B., Lucas C., Klein J., Silva J., Mao T., Oh J.E., Tokuyama M. (2020). Sex differences in immune responses that underlie COVID-19 disease outcomes. Nature.

[66] Zeng F., Dai C., Cai P., Wang J., Xu L., Li J. (2020). A comparison study of SARS-CoV-2 IgG antibody between male and female COVID-19 patients: A possible reason underlying different outcome between sex. J. Med. Virol..

[67] Sohrabi C., Alsafi Z., O’Neill N., Khan M., Kerwan A., Al-Jabir A., Iosifidis C., Agha R. (2020). World Health Organization declares global emergency: A review of the 2019 novel coronavirus (COVID-19). Int. J. Surg..

[68] Du Y., Zhou N., Zha W., Lv Y. (2021). Hypertension is a clinically important risk factor for critical illness and mortality in COVID-19: A meta-analysis. Nutr. Metab. Cardiovasc. Dis..

[69] Li X., Xu S., Yu M., Wang K., Tao Y., Zhou Y., Shi J., Zhou M., Wu B., Yang Z. (2020). Risk factors for severity and mortality in adult COVID-19 inpatients in Wuhan. J. Allergy Clin. Immunol..

